# Dual‐Modulated Vertically Stacked Transistors With Fully Laminated Plate‐Type Architecture Featuring Nanoscale Channel Length

**DOI:** 10.1002/advs.202519410

**Published:** 2026-02-03

**Authors:** Goeun Pyo, Su Jin Heo, Jeonggyun Jang, Hongki Kang, Byeongmoon Lee, Hyuk‐Jun Kwon, Jae Eun Jang

**Affiliations:** ^1^ Department of Electrical Engineering and Computer Science (EECS) Daegu Gyeongbuk Institute of Science and Technology (DGIST) Daegu Republic of Korea; ^2^ Department of Engineering Institute For Manufacturing University of Cambridge Cambridge UK; ^3^ Department of Biomedical Engineering Seoul National University College of Medicine Seoul Republic of Korea; ^4^ Department of Semiconductor Engineering Daegu Gyeongbuk Institute of Science and Technology (DGIST) Daegu Republic of Korea; ^5^ Institute of Medical and Biological Engineering, Medical Research Center Seoul National University Seoul Republic of Korea

**Keywords:** dual gate transistors, dual modulation, graphene transistors, nanoscale channel length, vertically stacked transistors, vertical thin film transistors, vertical transistors

## Abstract

A transistor with fully laminated plate‐type triode electrodes, source, drain and gate offers higher current density than a typical transistor design by allowing a 2D current path. Nanoscale transistors face challenges like off‐state leakage, so we introduce a new design using a laminated plate‐type architecture and a dual‐modulation strategy to improve performance and stability. Both top and bottom gates are used as active electrodes to fully control the channel's thickness. A micro‐hole patterned electrode is employed to enable effective gate field penetration into the channel, while a graphene electrode facilitates Fermi‐level modulation and improves field transfer. Furthermore, a leakage blocking layer is inserted to suppress unwanted carrier injection in the source and drain overlap regions. The device achieves low off‐state current of ≈10^−12^ A and an on/off‐current ratio exceeding 10^6^ at *V*_DS_ of 3 V. It also delivers high output currents under low‐voltage operation (1 mA cm^−2^ at 0.1 V and 50 mA cm^−2^ at 1 V). Despite a nanoscale channel length, the device maintains near‐zero *V*_TH_. The fully encapsulated channel shows strong reliability against bias stress and light. This work shows that a laminated vertical design with dual‐gate control effectively enhances the stability of nanoscale transistors, highlighting their potential for next‐generation low‐power logic, memory, and flexible electronics.

## Graphical abstract

A new vertical transistor design with a dual‐modulation strategy is proposed to overcome the limitations of conventional transistors. This architecture uses a micro‐hole patterned electrode and a graphene electrode to enhance channel control and incorporates a leakage blocking layer to suppress off‐state current. The device achieves a low off‐state current of ≈10^−12^A and a high on/off ratio exceeding 10^6^.

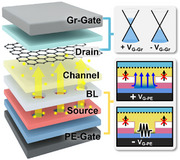

## 1 Introduction

The rapid development of next‐generation electronic device industries is driving an increasing demand for transistor architectures that offer both high performance and high reliability. As integration density continues to rise, conventional planar transistor designs face physical and process limitations, leading to higher power consumption and degraded electrical characteristics [1, 2, 3, 4]. As an alternative, vertically stacked transistors composed of laminated source, channel, drain, and gate electrodes have been proposed. This architecture enables the realization of nanoscale channel lengths through simple stacking, achieving high integration density and high current drivability under low operating voltages [5, 6]. because current flows along the 2D plane between plate‐type source and drain electrodes. In addition, the device is less affected by crack formation in the channel under mechanical deformation, making it suitable for flexible electronics [7, 8, 9].

However, vertically stacked transistors with fully laminated layers still face inherent structural challenges. Source (or drain) electrode located between the gate insulator and the channel induces gate field shielding, which severely limits an electrical controllability of channel layer. This field shielding issue is one of the most fundamental problems in vertical structures. To overcome it, some approaches have been suggested to physically or materially modify the electrodes causing the field shielding. For example, methods reported include forming extremely thin electrodes and partially oxidizing them to transmit the gate electric field via an oxide layer, introducing micro‐patterns into the electrode to allow field penetration through open areas, or applying carbon‐based electrodes like carbon nanotubes (CNTs) or graphene with a low density of states to modulate the Fermi level of the electrode itself, thereby enhancing field transmission efficiency [10, 11, 12, 13, 14, 15, 16, 17, 18, 19, 20]. However, in these vertical structures, the short channel length exacerbates the short‐channel effect. This issue is particularly pronounced in inorganic‐based channels designed for high output, due to their high mobility and low resistance characteristics. This causes a significant negative shift in the threshold voltage, making stable operation difficult. Additionally, leakage current increases in the off‐state due to overlapping source and drain channels or the inherently short channel length. Therefore, fundamentally resolving these channel control and leakage current issues is essential for vertically stacked transistors to become mainstream technology.

To overcome these limitations, this study introduces a dual‐modulation strategy employing structural and material modification. Unlike conventional dual‐gate transistors in which the two gates modulate the channel in essentially the same electrostatic manner, the proposed structure incorporates two heterogeneous field‐modulation pathways, thereby motivating the term dual modulation. Both the top and bottom gates are employed as controllable electrodes to enable more precise and stable channel control. Unlike single‐gate vertical transistors, the dual‐modulation architecture allows more delicate electrical controllability of the channel conductance, thereby alleviating short‐channel effects and greatly improving on/off operation stability [21, 22]. For electrostatic control, a micro‐hole patterned source electrode is suggested to facilitate gate field penetration into the channel, while a graphene drain electrode is used to enable Fermi‐level modulation and efficient field transfer. Furthermore, a leakage blocking layer is incorporated to suppress unwanted carrier injection in source–drain overlap regions that are not effectively modulated by the gate [23, 24].

Through these structural optimizations, the proposed device achieves low off‐state current, high on/off ratio, and stable threshold voltage control simultaneously. It also delivers high current drivability under low‐voltage operation, demonstrating its potential for high‐speed and low‐power applications. In addition, the channel layer is fully encapsulated by laminated electrodes (drain and source), providing strong immunity against external stresses such as negative bias stress and light illumination, which ensures reliable device operation without additional passivation processes [25, 26].

## 2 Results and Discussions

**FIGURE 1:**
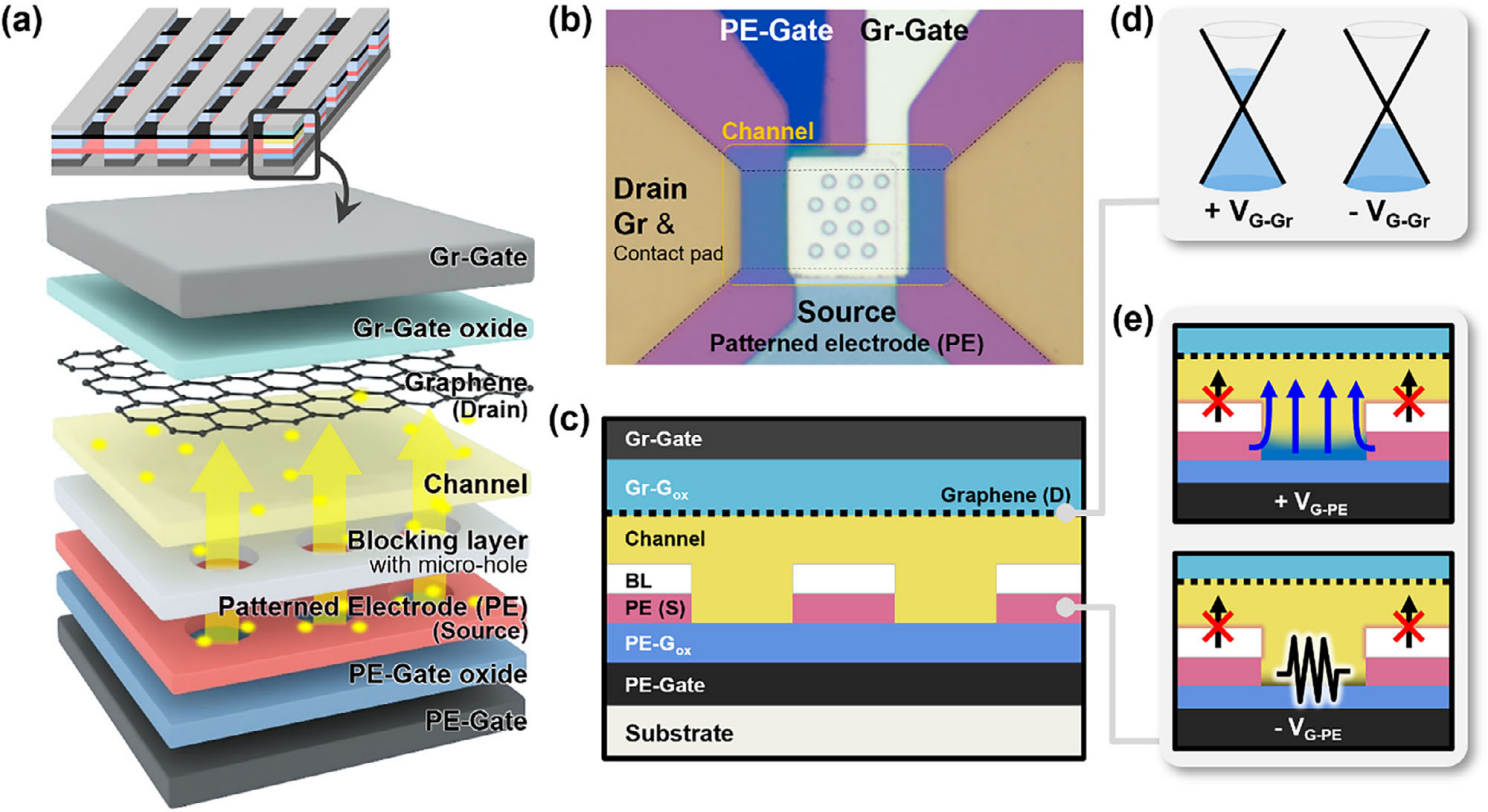
(a) Conceptual integration schematic and stacked components of a dual‐modulated vertical transistor. (b) Optical microscope image of the dual‐gate vertical transistor. (c) Cross‐sectional schematic image of dual‐modulated vertical transistor. (d) Schematic image of the graphene electrode working mechanism. (e) Schematic image of the patterned electrode side gate modulation.

Figure 1a illustrates the integrated design approach and layer stacking structure of the dual‐modulated vertical transistor. The dual‐modulated vertical transistor is simply stacked with fully laminated plate‐type electrodes including the PE‐gate, the PE‐gate oxide, the source with patterned electrode (PE), the channel, the drain formed by graphene (Gr), the Gr‐gate oxide, and the Gr‐gate, enabling electrostatic control from both sides. To block unwanted leakage currents that occur in the overlapping regions of the source and drain electrodes, a blocking layer (BL) that matches the shape of the patterned source electrode is placed on top of the source. The structural details of each layer of the vertical transistor are as follows. The PE‐gate is made of 50 nm of ITO. The PE‐gate oxide is made of 20 nm of HfO_2_, and the source electrode, designed as a metal structure with a patterned array of 7 µm holes, is made of 50 nm of ITO. The blocking layer is 50 nm of SiO_2._ The channel material is 40 nm of IGZO, which was chosen as it is a representative oxide semiconductor offering high electron mobility, excellent film uniformity, and stable electrical characteristics even under low‐temperature processing. These features made it particularly suitable for the proposed low‐temperature, vertically stacked structure [18]. The drain electrode is a monolayer graphene. The Gr‐gate oxide is 20 nm of HfO_2,_ and the Gr‐gate is 100 nm of aluminum. The source electrode includes holes, which transmit the gate field to the channel layer through these holes. To suppress unwanted leakage currents generated in the overlapping areas of the patterned source and drain electrodes, an insulating leakage blocking layer was placed directly above the patterned source. The layer features hole patterns that match those of the underlying source electrode. Without the blocking layer, the overlapping region between the source and drain electrodes is not influenced by the gate field and therefore retains a high carrier concentration that cannot be modulated by the PE‐gate as shown in Figure S1. This results in a significant off‐state leakage current and a poor on/off ratio. By introducing the blocking layer, vertical carrier transport through this uncontrolled region is effectively suppressed, which reduces the off‐state current and improves the overall switching performance of the device. Figure 1b shows a microscopic image of the dual‐gate vertically stacked transistor. The layers described above are sequentially stacked, and contact is made on both sides to verify the electrical properties and transfer characteristics of the graphene electrode. The hole structure is present in both the patterned electrode and the blocking layer. Figure 1c shows a cross‐sectional schematic diagram of the device, while Figure 1d,e illustrates the mechanisms by which each gate modulates the channel layer. The Gr‐gate located on the graphene electrode side adjusts the Fermi level of the graphene based on the applied field, resulting in better on‐off modulation. When a positive voltage is applied to the Gr‐gate, n‐type doping of graphene occurs, whereas a negative voltage results in p‐type doping effect. This phenomenon is illustrated in the energy band diagram shown in Figure 1d, and the transfer characteristics of the graphene used in this study can be seen in Figure S2 [18, 28]. The PE‐gate transfers the gate field to the channel layer through the hole regions of the patterned electrode. As shown in Figure 1e, the overlapping region of the source and drain electrodes is insulated by the blocking layer, which prevents direct charge transport, whereas, carriers move through the hole regions controlled by the gate. When a positive voltage is applied to the PE‐gate, carriers accumulate in the hole regions, reducing the resistance and facilitating carrier movement. In contrast, when a negative voltage is applied to the PE‐gate, the electrons in the channel layer are depleted, increasing the channel layer film resistance. These phenomena enable the operation of the dual‐modulated vertical transistor through the complementary modulation of the two gates.

**FIGURE 2:**
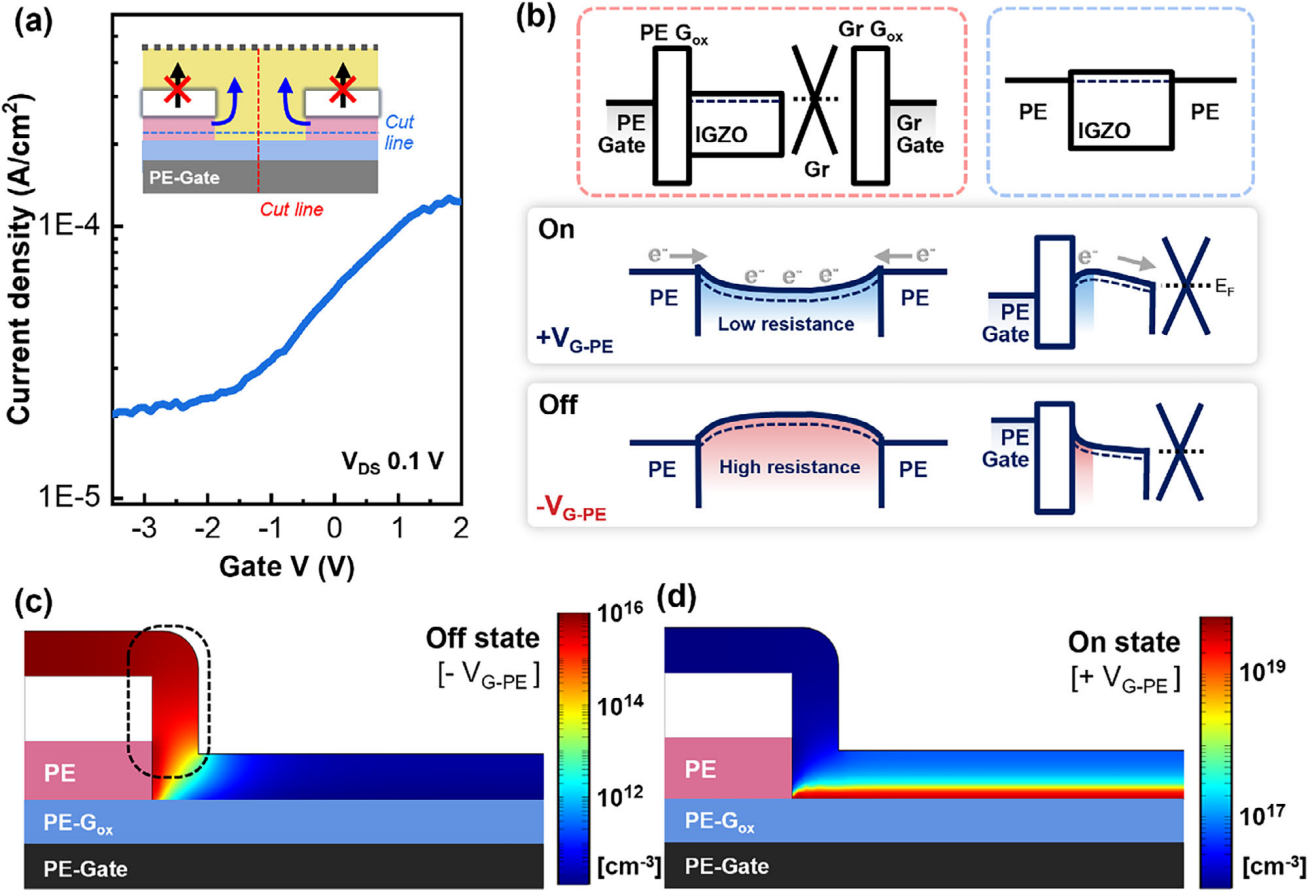
(a) Transfer curve with PE‐gate modulation only. (inset: carrier movement path) (b) Schematic image of the on‐off working principle of the vertical transistor with PE‐gate modulation. (c) Cross‐sectional simulation of the off‐state carrier concentration and (d) on‐state carrier concentration when only the patterned electrode‐side gate is operating.

To analyze the dual‐modulation mechanism, it is essential to first understand each single‐gate operation mechanism. For this purpose, we performed electrical characterization and carrier concentration simulations for single‐gate operation. Additionally, the other gate was kept at a fixed bias, serving as a secondary controllable electrode to clarify the modulation characteristics. Figure 2a shows the transfer curve obtained by sweeping only the PE‐gate, with a drain voltage of 0.1 V applied. The on/off operation is controlled via the hole, and a leakage current blocking layer is introduced to minimize the region not controlled by the gate voltage. Despite the nanoscale channel length, modulation is observed as the gate voltage changes.

This on‐off modulation mechanism is illustrated in the energy band diagram shown in Figure 2b. The top of Figure 2b presents the flat band structure of the vertical transistor and the energy band structure at the micro‐hole region, corresponding to the red and blue cut lines, respectively, indicated in the schematic diagram of Figure 2a. When a positive voltage is applied to the PE‐gate, this bending causes electrons to move from the patterned electrode, i.e., source electrode into the channel region, lowering the channel resistance. The gate field induces energy band bending in the channel region, where carriers accumulate. As electrons accumulate, resistance decreases, causing carriers to flow from the source to the drain electrode. Conversely, when a negative voltage is applied to the PE‐gate, the energy band of the channel region bends upward, increasing the resistance of the thin film. As a result, carriers do not accumulate in the channel layer of the micro‐hole region, restricting electrons transport and leading to the off‐state. Figure 2c,d shows the carrier concentration and distribution in the off and on‐states. The simulation was conducted to analyze the carrier concentration in the channel region using COMSOL Multiphysics. A negative or positive voltage was applied to the PE‐gate. In the off‐state, the carrier concentration in the micro‐hole region decreases significantly. However, due to the inability of the gate field to reach upper area of the patterned electrode (the black dotted area in Figure 2c), relatively high carrier concentrations remain in these regions. In other words, a high off‐state leakage current occurs due to the high carrier concentration in gate‐uncontrolled regions in the off‐state. The leakage current is induced by the carrier movement path shown in the inset of Figure 2a. Under the vertical electric field generated by the drain voltage (V_DS_), carriers move from the patterned electrode to the channel layer and then to the graphene electrode at the off‐state. In the on‐state, the hole region exhibits high carrier concentration, whereas the regions above the patterned electrode display relatively low carrier distribution, highlighting the field‐shielding effect that motivates the need for dual modulation. Furthermore, the on‐current is not relatively high even when the PE‐gate is in the on‐state. This is because modulation is limited to only hole‐based control, the low on‐current also results from the material properties of graphene. For these reasons, the achievable on/off ratio during single‐gate operation remains below 10.

To overcome the limitation of only PE‐gate drive and identify a dual‐modulation approach for improving on/off characteristics, we analyzed the electrical properties by operating the Gr‐gate, which modulates the graphene electrode, as an assist gate. Graphene has a unique energy band structure derived from its hexagonal lattice, with a Dirac point where the conduction and valence bands meet. Near the Dirac point, the density of states decreases, and when the Fermi level of graphene is at the Dirac point, the density of states is minimized, resulting in maximum resistivity. The Fermi level of graphene can be controlled through the gate. A positive gate voltage induces n‐type doping effect on graphene, while a negative gate voltage induces p‐type doping effect. When only the PE‐gate is operated, no doping effect occurs in the graphene and its Fermi level remains near the Dirac point, resulting in high electrode resistance.

**FIGURE 3:**
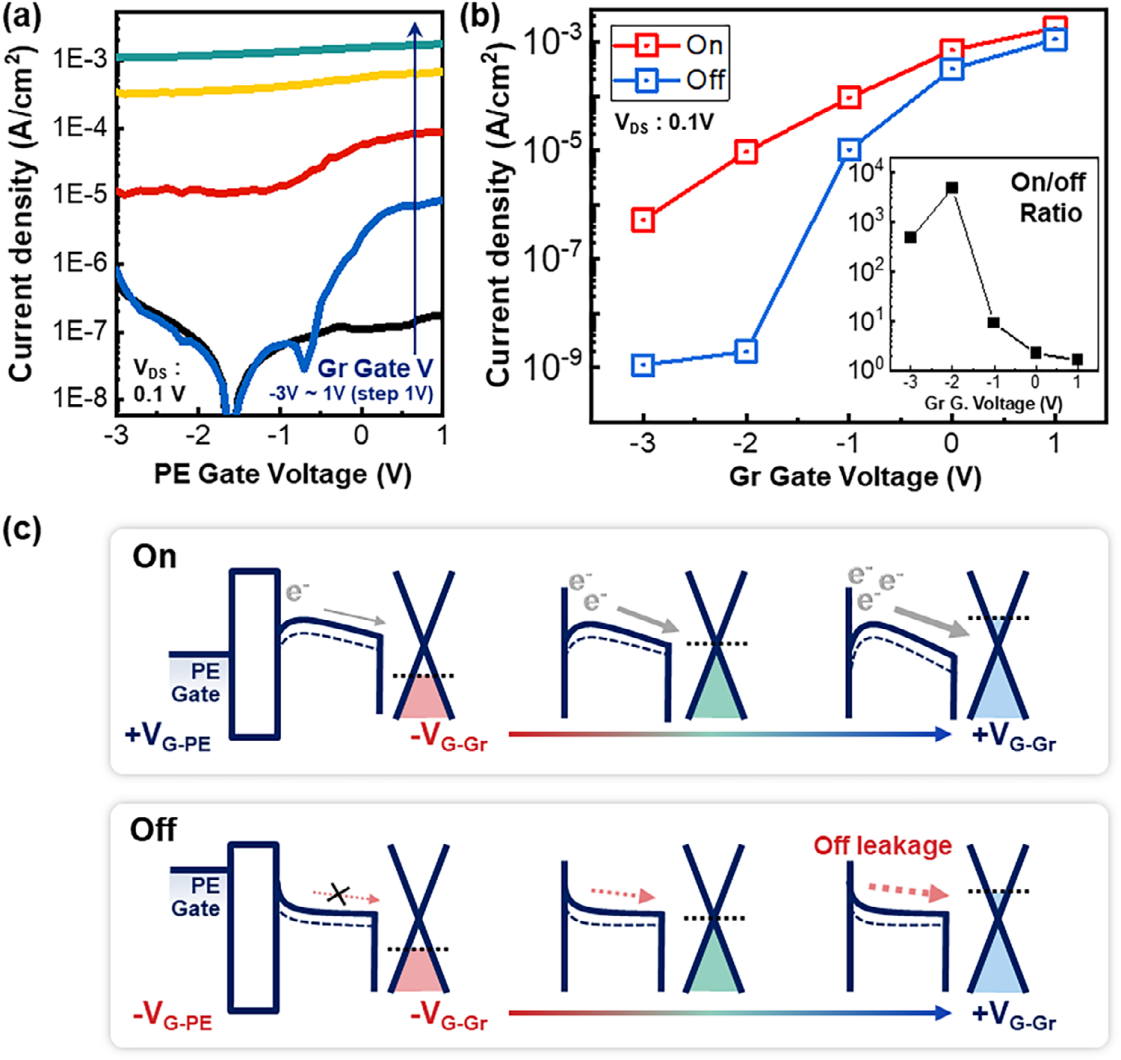
(a) Transfer curves of the PE‐gate with increasing Gr‐gate bias from ‐3 to 1 V in 1 V steps. The drain voltage was fixed at 0.1 V. (b) On and off current depending on the Gr‐gate. (inset: on‐off ratio) (c) Schematic image of the on‐off working principle with assisted Gr‐gate bias.

Figure 3a shows the transfer curve measured by sweeping the PE‐gate while applying a fixed voltage to the Gr‐gate. The voltage applied to the Gr‐gate ranged from ‐3 to 1 V, with intervals of 1 V. When the Gr‐gate voltage is negative, both the on and off‐currents remain low. This is because applying a negative voltage to the Gr‐gate, coupled with graphene's weak electrical screening effect, causes the gate field to increase device resistance through two mechanisms. First, the gate field penetrates the graphene, creating a depletion region and increasing resistance [29]. Second, the gate field lowers the Fermi level in graphene, inducing a p‐type doping effect. This raises the Schottky barrier between graphene and the IGZO channel, increasing the interfacial resistance. Conversely, when the Gr‐gate voltage increases to a positive voltage, both the on‐current and off‐current increase due to the reduction in channel resistance and the relaxation of the barrier. Figure 3b shows the on‐ and off‐currents and the on/off ratio of the transfer curve obtained by sweeping the PE‐gate at different Gr‐gate voltages. The assisting gate bias from the Gr‐gate clearly influences the PE‐gate transfer characteristics, demonstrating that the Gr‐gate plays a critical role in dual‐gate operation and is essential for achieving dual modulation. Graphene present across the entire active region influences the entire channel layer, enabling higher output currents by simultaneously controlling both the channel resistance and the intrinsic resistance of the graphene itself. Figure 3c demonstrates this mechanism. When PE‐gate is on‐state, an increase in the Gr‐gate voltage raises the Fermi level of graphene, lowering the resistance of the thin film and enhancing carrier transport. This results in an increase in the output current. Conversely, when the PE‐gate is off‐state, an increase in the Gr‐gate voltage reduces the graphene resistance and channel resistance, resulting in higher off‐state leakage currents. These graphene–IGZO junction characteristics can be confirmed in Figure S3. Therefore, both the PE‐gate and Gr‐gate produce high output current when a positive gate voltage is applied, and the lowest off‐current is achieved when both gates have a negative gate voltage. Based on this, the on‐off characteristics can be enhanced by dual modulation.

**FIGURE 4:**
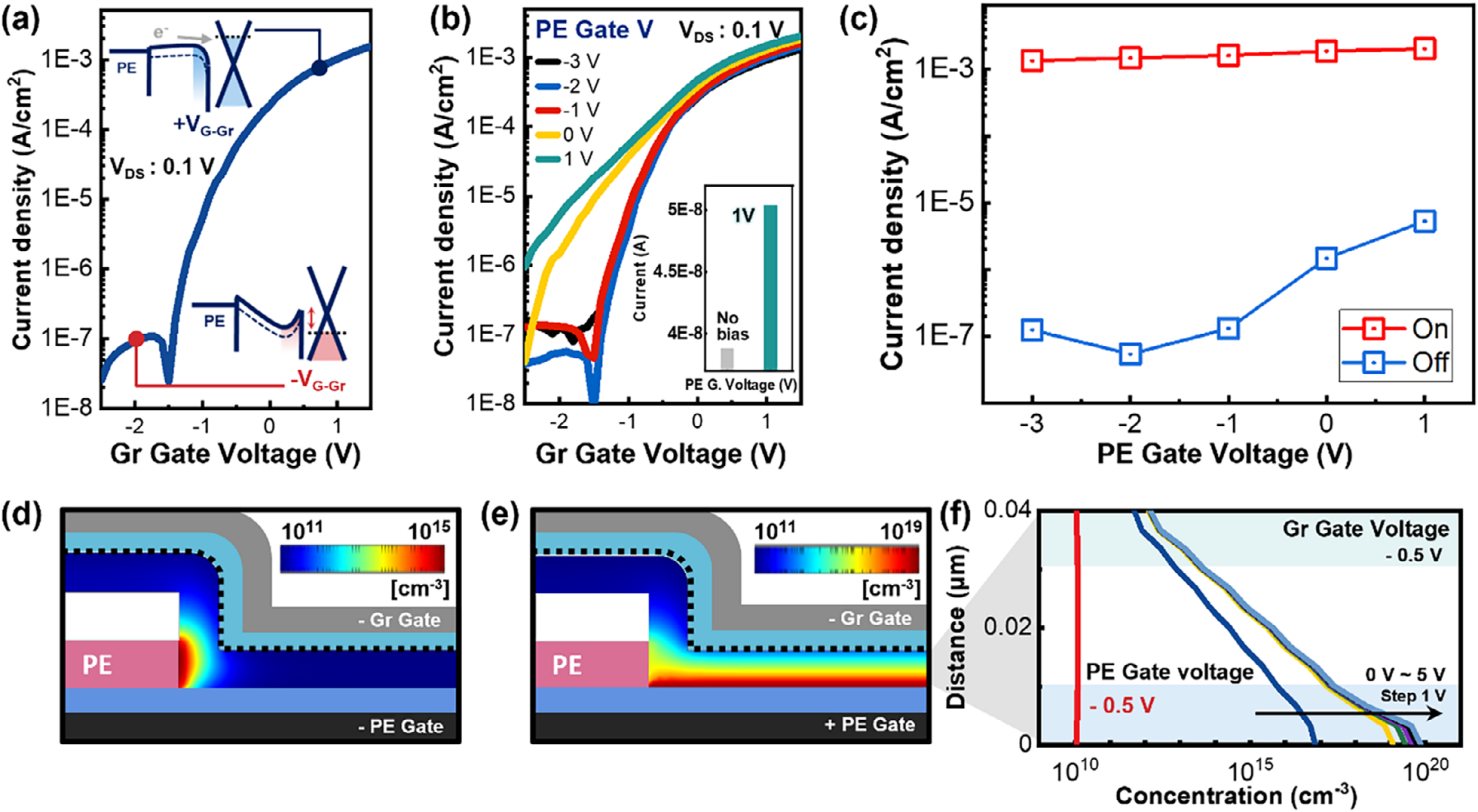
(a) Transfer curve with Gr‐gate modulation only. (inset: schematic image of on‐off working mechanism) (b) Gr‐gate transfer curves with increasing PE‐gate bias from ‐3 to 1 V. (*V*_DS_ = 0.1 V) (c) on and off current depending on the PE‐gate. (d) Cross‐sectional carrier distribution simulation of the two gate bias: both off‐state, (e): PE‐gate on‐state. (f) Simulation of carrier concentration as a function of channel depth with various PE‐gate bias at ‐0.5 V Gr‐gate voltage.

To analyze the dual modulation principle from the Gr‐gate perspective, electrical characteristics were performed. Figure 4a shows the transfer curve obtained by sweeping only the Gr‐gate with a drain voltage of 0.1 V applied. Compared with single modulation by the PE‐gate, this curve demonstrates reduced off‐state current and enhanced on‐state current. The low off‐state current is attributed to the reduced overlap area by the blocking layer above the patterned electrode and gate‐controllable graphene present across the entire active area. The mechanism of the energy band operation is shown in the inset of Figure 4a. When a negative bias is applied to the Gr‐gate, the Fermi level of graphene decreases, forming a Schottky barrier between IGZO and graphene, which suppresses carrier transport and defines the off‐state condition within the modulation scheme. In the on‐state, the Fermi level of the graphene increases, reducing the resistance at the IGZO interface. Carrier accumulation further reduces the thin‐film resistance, enabling on‐state operation and demonstrating how graphene actively participates in channel control. Figure 4b shows the transfer curve obtained by sweeping the Gr‐gate as the main gate while sequentially applying fixed voltages ranging from ‐3 to 1 V to the PE‐gate. As the voltage of the PE‐gate increases and the Gr‐gate is at a negative voltage (off‐state), an increase in the off‐current and a deterioration in the subthreshold slope (SS) are observed. This is due to carrier accumulation in the channel near the patterned electrode, which causes leakage current even in the off‐state. The inset of Figure 4b compares the on‐current when no voltage is applied to the PE‐gate with the on‐current when 1 V is applied. Applying 1 V to the PE‐gate results in approximately a 30% increase in current compared to the case with no voltage applied. The variation of the on‐off current with respect to the PE‐gate voltage is shown in Figure 4c. To investigate the carrier concentration distribution when each gate operates independently, simulations were performed. Figure 4d,e shows how carriers are distributed within the channel when the Gr‐gate is in the off‐state with a negative voltage, and PE‐gate is at either a negative or positive voltage. When both gates are at negative voltages, the carrier concentration decreases in all regions of the channel except near the patterned electrode. In contrast to the off‐state of only the PE‐gate shown in Figure 2c, the channel region above the blocking layer and the patterned electrode also shows a reduced carrier concentration, breaking the transport path from the patterned electrode to the graphene. When the Gr‐gate is off‐state and the PE‐gate is on‐state, carriers accumulate in the hole regions of the patterned electrode. These accumulated carriers cause leakage current under the vertical field generated by *V*_DS_. The red graph in Figure 4f shows the channel concentration when both gates are negative bias, where the carrier concentration is uniformly low. As the voltage of the PE‐gate increases, the carrier concentration in the channel region near the patterned electrode also increases. Although the carrier concentration decreases near the Gr‐gate, it remains higher than when both gates are off‐state. These changes in the carrier concentration distribution are one of the main reasons for the increased off‐state leakage observed in Figure 4b. Additionally, the carrier distribution for all gate combinations can be seen in Figure S4.

**FIGURE 5:**
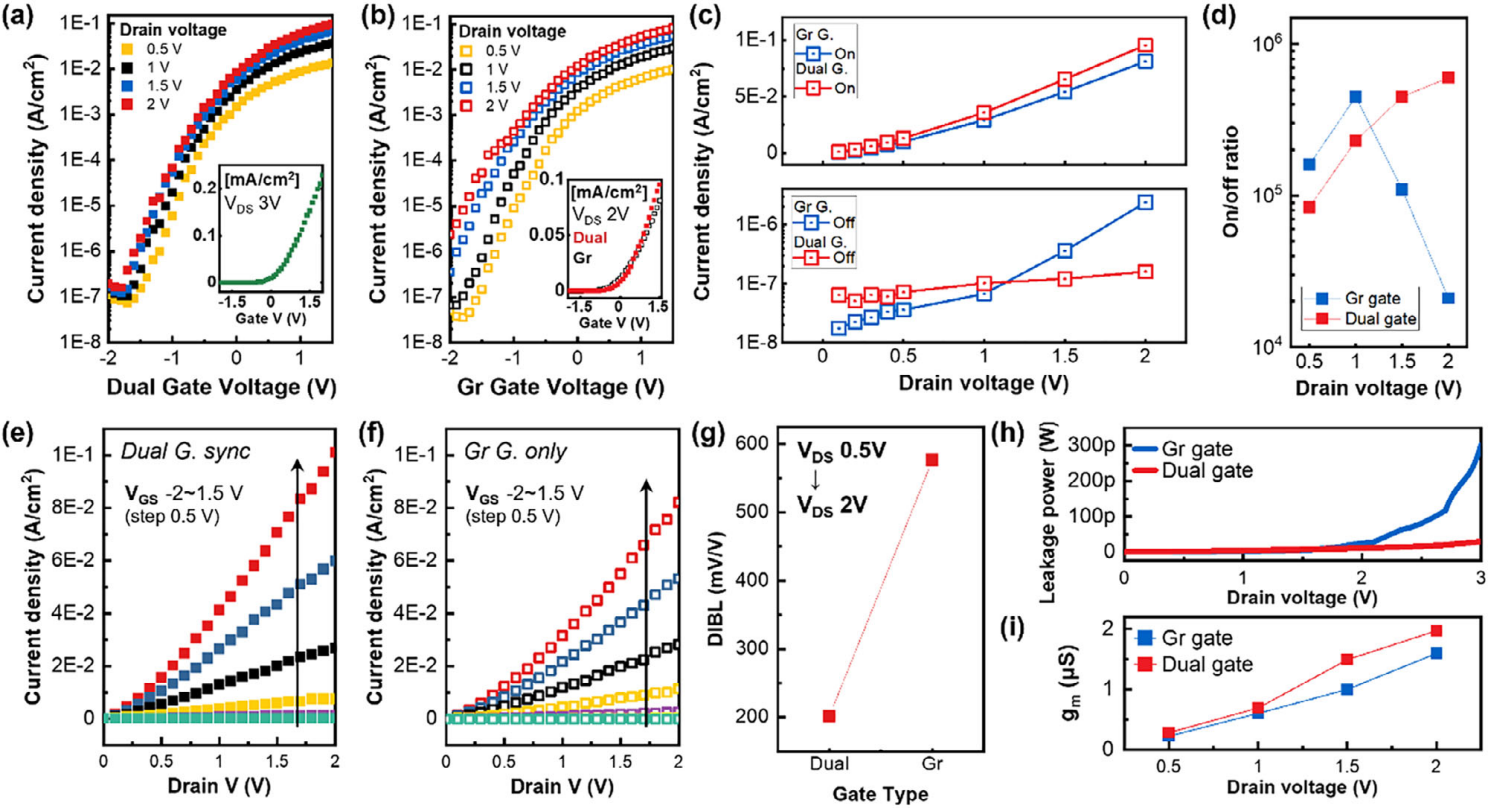
(a) Transfer curves as a function of drain voltage in synchronized dual gate modulation. (inset: V_DS_ 3 V). (b) Transfer curves as a function of drain voltage under single Gr‐gate modulation. (inset: linear scale transfer curves of both gate modulation). (c) On and off current as a function of drain voltage for single Gr‐gate modulation and synchronized gate dual modulation. (d) On/off ratio with various drain voltages. (e) Output curves under synchronized dual gate modulation. (f) Output curves single Gr‐gate modulation. (g) Comparison of DIBL by gate type. (h) Off‐state leakage power for single Gr‐gate modulation and synchronized dual gate modulation. (*V*_GS_ ‐2 V). (i) Comparison of the maximum transconductance (*g*_m_) extracted from single Gr‐gate modulation and synchronized dual gate modulation.

With both gates biased at negative voltages, the off‐state current remains lower, whereas applying positive voltages to both gates leads to an increased current. Therefore, synchronizing the modulation of the two gates, rather than operating them independently, achieves both low off‐state current and high output current. This can be regarded as a significant advantage of our proposed design, because although the structure contains two gates, it only requires the application of the same driving voltage. As a result, no additional interconnections or separate driving circuits are needed, which simplifies the overall system configuration. Figure 5a,b shows the transfer curves obtained when the dual gates are synchronized and when only the Gr‐gate is active, respectively. At low drain voltages, both devices exhibit low off‐state current, but the dual‐gate synchronized configuration delivers slightly higher on‐current. As the drain voltage increases, the off‐state current of the transistor with only the Gr‐gate increases by a factor of 10 for every 0.5 V increase in drain voltage. In contrast, the dual‐gate synchronized transistor maintains a low off‐state current with minimal variation. The inset of Figure 5a shows the transfer curve during dual modulation at a 3 V drain voltage. At this point, the leakage current is indeed in the 1 pA range. The difference between the on‐and off‐state currents is summarized in Figure 5c, which compares the operation with only the Gr‐gate active and with both gates synchronized (dual‐gate). Operating the dual gates in synchronization allows a larger portion of the channel to be effectively modulated, resulting in a higher on‐state output current. Importantly, this enhancement is achieved without additional power dissipation. For the dual‐gate vertical transistor, the off‐state current remains nearly constant regardless of changes in the drain voltage. In contrast, for the transistor with only the graphene‐side gate active, the leakage current increases as the drain voltage increases, since a single gate provides insufficient electrostatic control, whereas dual modulation extends gate influence across the entire channel thickness. Figure 5d presents the on/off current ratio as a function of drain voltage. While the device with only the Gr‐gate shows a rapid degradation of the on/off ratio at higher drain voltages, the synchronized dual‐gate modulation maintains a steadily increasing ratio, indicating superior robustness against drain‐induced effects. This behavior demonstrates that synchronized dual‐gate modulation ensures consistent switching performance even under high drain bias conditions. Figure 5e,f shows the output curves for the synchronized dual‐gate vertical transistor and only Gr‐gate transistor. As with the transfer curves, the transistor with synchronized gates for dual modulation provides higher on‐state current. As summarized in Table S1, this work demonstrates that synchronized dual‐gate operation enables lower off‐state current, higher on/off current ratio, and reduced gate operating voltage compared to other graphene‐based vertical transistors reported in recent research.[19, 20, 27, 32, 33, 34] Notably, the off‐state current remains nearly unchanged even as the drain voltage increases, indicating improved electrostatic control and robustness against drain‐induced leakage.

Figure 5g compares the drain‐induced barrier lowering (DIBL) extracted from the threshold‐voltage shift as a function of drain bias. The equations below show the definition and related equations of DIBL [30].

(1) $$\mathrm{DIBL}=-\frac{\Delta V_{\mathrm{th}}}{\Delta V_{d}}$$

Equation (1) defines DIBL as the variation in threshold voltage (*V*_th_) with increasing drain voltage (*V*_d_). A larger DIBL value indicates a greater reduction in threshold voltage with increasing drain bias, implying more pronounced short‐channel effects in the device.

(2) $$\mathrm{DIBL}\propto \frac{C_{d}}{C_{g}}$$

(3) $$C_{g}=\frac{\varepsilon_{\mathrm{ox}}}{t_{\mathrm{ox}}}\;A_{\mathrm{eff}},\left(A_{\mathrm{eff}}\approx \chi_{\mathrm{top}}A_{\mathrm{top}}+\chi_{\mathrm{bot}}A_{\mathrm{bot}}\right)$$

Equations (2) and (3) show the relationship between DIBL and capacitance. DIBL is proportional to the ratio between the drain capacitance (*C*_d_) and the gate capacitance (*C*_g_). A larger *C*_g_ relative to *C*_d_ enhances gate control and suppresses DIBL. At this time, Gate capacitance depends on the oxide permittivity (*ε*_ox_), oxide thickness (*t*_ox_), and the effective gate area (*A*_eff_). The *A*_eff_ includes the field‐transfer efficiency factors χ_top_ and χ_bot_ which reflect the structural and material characteristics of each electrode.

(4) $$\mathrm{DIBL}\propto \frac{C_{d}\;t_{\mathrm{ox}}}{\varepsilon_{\mathrm{ox}}\;A_{\mathrm{eff}}}$$

In conclusion, substituting the gate capacitance equation into the DIBL equation yields Equation (4). Increasing the *A*_eff_ and controlling the gate oxide layer are effective ways to minimize DIBL. In the dual‐gate vertical structure, the enlarged *A*_eff_ simultaneous actuation of both gates synergistically enhance electrostatic control over the channel. As a result, the drain‐induced influence is more effectively screened, leading to suppressed DIBL and improved threshold voltage stability. Compared to single‐gate operation, the dual modulated transistor offers a more robust response against drain bias variations.

This strengthened electrostatic control not only improves threshold voltage stability but also helps suppress off‐state leakage under high drain bias. The off‐state leakage power is defined as *P*_off_ = *V*_D_ * *I*_off_. As shown in Figure 5h, even when the drain voltage increases up to 3 V, the synchronized dual‐modulated device maintains P_off_ nearly constant, whereas the Gr‐gate only device exhibits a rapid increase. This indicates that dual modulation effectively suppresses drain‐induced leakage, leading to a significant reduction in standby power consumption. To accurately compare the gate modulation performance, the maximum transconductance (g_m_) values were extracted. As shown in Figure 5i, synchronized dual modulation enables higher g_m_ values without requiring additional power sources or control circuits, as both gates cooperatively couple to a larger portion of the channel and enhance the effective gate‐to‐channel capacitance. Additionally, the effect of the hole ratio of the patterned electrode on the electrical characteristics can be seen in Figure S5.

**FIGURE 6:**
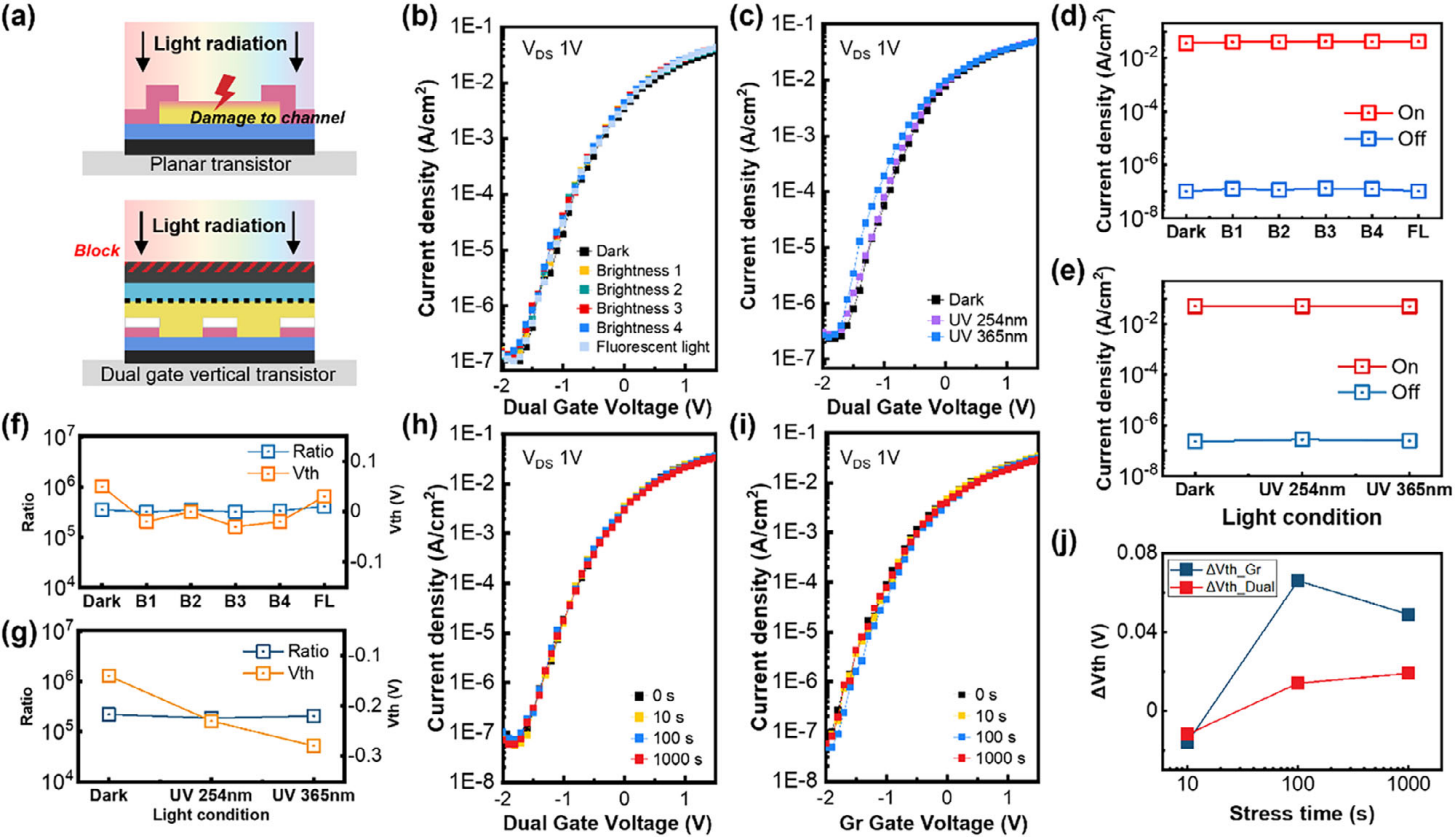
(a) Illustration of channel layer responses to light exposure depending on transistor structure. (b) Transfer curves of the dual‐modulated vertical transistor as a function of fluorescent and LED light intensity, and (c) as a function of UV power. (d) On‐ and off‐currents of the dual‐modulated vertical transistor as a function of fluorescent and LED light intensity, and (e) as a function of UV power. (f) On/off ratio and threshold voltage of the dual‐modulated vertical transistor as a function of fluorescent and LED light intensity, and (g) as a function of UV power. (h) Transfer curves of the dual‐modulated vertical transistor under negative gate bias stress over time. (i) Transfer curves of the single Gr‐gate modulation under negative gate bias stress over time. (j) Threshold voltage shift depending on stress time.

One of the significant advantages of the proposed dual‐gate vertical design is its self‐passivating structure that fully encapsulates the channel. This structural feature provides inherent protection against external stresses such as light exposure. Figure 6a shows a schematic comparison of a conventional transistor without passivation and a dual‐gate vertical transistor when exposed to light. In the conventional bottom‐gate transistor, the channel is exposed, allowing light to reach the active region and potentially cause device breakdown or output fluctuations due to light‐induced reactions. In contrast, the dual‐modulated vertical transistor features a fully encapsulated channel surrounded by gates, insulating layers and electrodes, which block light penetration and shield the channel. This configuration not only resists external perturbations without additional passivation but also enables reliable dual modulation of the channel. Similarly, planar transistors typically require careful storage or additional passivation to prevent channel degradation overtime. In contrast, the vertical structure inherently protects the channel, allowing the device to remain stable without additional post‐processing.

To confirm these characteristics, the stability of the dual‐gate vertical transistor was tested under LED light and UV light exposure. Figure 6b shows the transfer curve of the dual‐gate vertical transistor under ambient fluorescent light and varying intensities of LED light. The drain voltage during these measurements was 1 V. The applied brightness levels (steps 1–4) correspond to approximately 3, 6, 12, and 15 mW, respectively, as measured with an external power meter, and were converted to a maximum intensity of approximately 5.3 mW cm^−2^ based on the illuminated area. These conditions fall within the standard illumination range (a few to several tens of mW cm^−2^) typically used in optoelectronic device testing. All measurements were performed under constant illumination at each step to ensure reliability. The variations of on‐off current, on‐off ratio, and threshold voltage (*V*_th_) under different light intensities are summarized in Figure 6d,f. Even as light intensity increased, the electrical characteristics showed negligible changes confirming stable modulation behavior under illumination. The device was further tested under UV light, which has higher power than LED or fluorescent light, to examine its electrical stability. Figure 6c shows the electrical characteristics in the absence of light and under UV light at wavelengths of 254 and 365 nm. Measurements were taken continuously during UV exposure. Figure 6e,g summarize the on–off current, on‐off ratio and *V*_th_ shifts under different UV wavelengths. Although higher UV power induced a slight negative shift in *V*_th_, the on/off current and ratio remained virtually unchanged, indicating that dual modulation maintains robust switching even under severe light stress. The *V*_th_ shift was minimal even under UV exposure, indicating strong resistance to light‐induced effects. This minor shift in threshold voltage is attributed to the laboratory‐scale fabrication margin. Although the channel is structurally shielded by the top metal layer, a small portion of the peripheral region may be exposed during lithographic patterning, allowing limited light‐induced carrier generation. Moreover, since the photon energy of visible light is below the bandgap of IGZO, it does not induce photoexcitation, while UV light with higher energy can generate photoexcited electrons, leading to a slight negative *V*_th_ shift. Despite this, the on/off performance remained stable, confirming robust dual‐gate modulation. Additional measurements on multiple devices under LED and UV illumination were conducted to assess reproducibility, and similar trends were consistently observed, as shown in Figure S6.

After confirming the light resistance of the dual‐gate vertical transistor, its performance under negative gate bias stress was analyzed to determine whether it maintains stability under such conditions. To make accurate comparisons, the performance of the device with only the Gr‐gate active was compared to that with both gates synchronized. Under synchronized dual modulation, applying ‐1.5 V gate stress for 1000 s produced negligible *V*_th_ variation and stable on/off currents. In contrast, single Gr‐gate modulation led to noticeable output fluctuations, highlighting the superior stress resistance of dual modulation. Figure 6j summarizes the time evolution of the *V*_th_ shift, clearly showing that Gr‐gate‐only modulation is more susceptible to stress, whereas synchronized dual modulation effectively suppresses stress‐induced instability. Overall, these findings highlight that synchronized dual‐gate modulation not only enhances electrical performance but also contributes to improved operational stability under negative gate bias stress. Based on a log‐time extrapolation of the measured short‐term stress data, the projected threshold voltage shift for the dual‐gate device is estimated to be approximately 0.10 V after 5 years and approximately 0.11 V after 10 years of continuous operation [31]. In contrast, the Gr‐gate only device is expected to exhibit a significantly larger *V*_th_ shift of approximately 0.23 V after 5 years and approximately 0.24 V after 10 years under the same extrapolation conditions. Although these projected values are derived from extrapolated trends rather than direct long‐term measurements, the markedly smaller *V*_th_ drift observed in the dual‐gate device suggests a more favorable long‐term operational stability compared to the single‐gate counterpart. The extrapolated results indicate that the increase in threshold voltage shift from 5 to 10 years is minimal for both devices, particularly for the dual‐gate configuration. This behavior implies that degradation beyond the initial stress regime is likely to follow a gradual saturation trend rather than exhibiting accelerated deterioration during prolonged operation. Nevertheless, these long‐term projections are estimated values based on the assumption that the log‐time dependence observed during short‐term stress remains valid over extended periods. Therefore, the possible involvement of additional trap generation, interface degradation, or alternative degradation mechanisms during actual long‐term operation cannot be fully excluded. Accordingly, the extrapolated results presented here should not be interpreted as an absolute lifetime guarantee, but rather as an indication that synchronized dual‐gate modulation offers improved long‐term stability compared to conventional single‐gate operation under conservative stress conditions.

## 3 Conclusion

In this study, we demonstrated a dual‐modulated vertical transistor with a leakage‐blocking layer, fabricated through a simple stacked process without the need for high‐resolution alignment or costly techniques, even at nanoscale channel lengths. The device architecture, featuring a fully encapsulated channel, micro‐hole patterned source electrode, and a graphene drain, enabled complementary gate field modulation across the entire channel, effectively suppressing leakage currents and ensuring stable threshold voltage control. As a result of dual modulation, the transistor exhibited a low off‐state current of ≈1 pA at 3 V, an on/off ratio exceeding 10^5^, and high output currents of ≈1 mA cm^−2^ at 0.1 V and ≈50 mA cm^−2^ at 1 V, while maintaining a threshold voltage within ±0.5 around 0 V.

Furthermore, the device structure proposed in this study was designed not only for excellent electrical performance but also considering practical manufacturability in large‐area fabrication and vertical stacking processes. In the proposed architecture, the most alignment‐sensitive process steps require only sub‐micron‐level alignment accuracy. Importantly, the structure does not rely on repeated nanoscale alignment between vertically stacked functional layers, thereby mitigating the accumulation of alignment errors in wafer‐level fabrication. Other process steps do not demand extremely high alignment precision, providing a structural advantage favorable for large‐area process application. Moreover, since all processes are performed within a low‐temperature range, no degradation in the crystallinity of the channel material or additional thermal damage occurs even during repeated vertical stacking processes. Interlayer variations that could accumulate due to the micro‐hole structure during multi‐layer stacking are expected to be effectively managed through precise thickness control of the intermediate insulating layer and planarization processes such as chemical mechanical planarization (CMP). These manufacturing considerations suggest that the proposed dual‐modulation vertical transistor structure can serve as a scalable platform for high‐density 3D integrated circuits and large‐area system integration.

## 4 Experimental Section

### 4.1 Fabrication Process

The fabrication process of the laminated plate‐type vertically stacked transistor was as follows. A transparent glass substrate was used as the substrate. The transistor consisted of top and bottom gates, starting with the bottom gate. A 50 nm thick PE‐gate of indium tin oxide (ITO) was deposited via RF magnetron sputtering, patterned, and then etched with diluted HCl. A 20 nm thick PE‐gate oxide of HfO_2_ was then deposited using thermal ALD with TEMAHF and H_2_O as precursors. Approximately 200 cycles were applied to achieve the 20 nm thickness. The source electrode, a patterned electrode (PE) with a micro‐hole array, was formed from a 50 nm ITO layer by deposition, patterning, and etching. A 50 nm thick blocking layer was deposited using PECVD and patterned to align precisely with the source's microholes. Following this, the channel layer of 40 nm thick indium gallium zinc oxide (IGZO) was deposited via RF magnetron sputtering, patterned, and etched with diluted HCl, then annealed at 300°C for 1 h to improve its properties. A monolayer graphene film, grown by CVD, was transferred onto the IGZO to serve as the drain electrode. The graphene was patterned by photolithography and etched using RIE with oxygen plasma. The active area where the source and drain overlap is 50 µm × 50 µm. 40 nm thick nickel layer was evaporated and patterned via a lift‐off process to create contact pads for the graphene. A 20 nm thick Gr‐gate oxide of HfO_2_ was deposited using thermal ALD, and a 100 nm thick Gr‐gate of aluminum (Al) was then evaporated. Since the ALD chamber temperature (170°C) is significantly lower than the post‐annealing temperature of the previously deposited IGZO channel (300°C), the HfO_2_ deposition step does not affect the crystallinity or electrical characteristics of the IGZO layer. Finally, RIE was used to open the contact areas, completing the device.

### 4.2 Monolayer Graphene Transfer Process

The graphene used in the device is a monolayer graphene grown on a copper foil by chemical vapor deposition (CVD), and it exists on both sides of the foil. To prepare the film for transfer, one side of the graphene was protected with a PMMA layer, while the graphene on the opposite side was removed using a reactive‐ion etching (RIE) system. The copper foil was then etched away using an ammonium persulfate (APS) solution, isolating the graphene film. Following the copper etching, the PMMA‐coated graphene was rinsed with deionized (DI) water to remove any residual copper etchant. The rinsed graphene was then transferred onto the substrate. Finally, the protective PMMA layer was removed with acetone, completing the transfer process.

## Conflicts of Interest

The authors declare no conflicts of interest.

## Supporting information




**Supporting File 1**: advs74203‐sup‐0001‐SuppMat.docx.

**Supporting File 2**: advs74203‐sup‐0002‐Data.zip.

## Data Availability

The data that support the findings of this study are available from the corresponding author upon reasonable request.
