# Supplementary material for: Dual‐Modulated Vertically Stacked Transistors With Fully Laminated Plate‐Type Architecture Featuring Nanoscale Channel Length
Source: Adv Sci (Weinh). 2026 Feb 3;13(20):e19410. doi: 10.1002/advs.202519410 (PMC13067813; doi:10.1002/advs.202519410)
Supplement: Supplementary file 1 — Supporting File 1: advs74203‐sup‐0001‐SuppMat.docx. [file ADVS-13-e19410-s001.docx]

Supporting Information

**Dual-Modulated Vertically Stacked Transistors with Fully Laminated Plate-type Architecture Featuring Nanoscale Channel Length**

Goeun Pyo, Su Jin Heo, Jeonggyun Jang, Hongki Kang, Byeongmoon Lee, Hyuk-Jun Kwon and Jae Eun Jang*


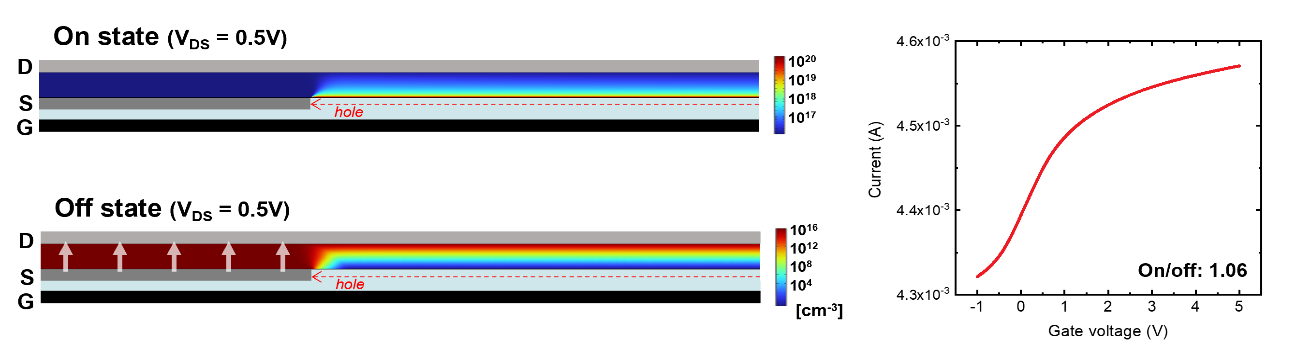


Figure S1. Simulation of carrier distribution and transfer characteristics in the vertical transistor without the blocking layer.


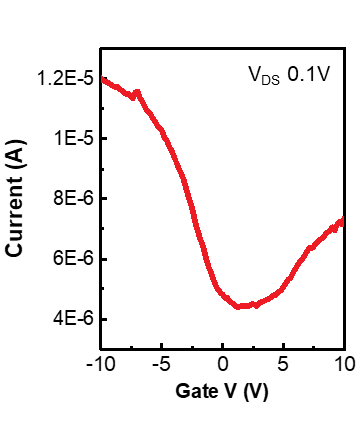


**Figure S2.** Graphene transistor transfer curve at drain voltage 0.1V.


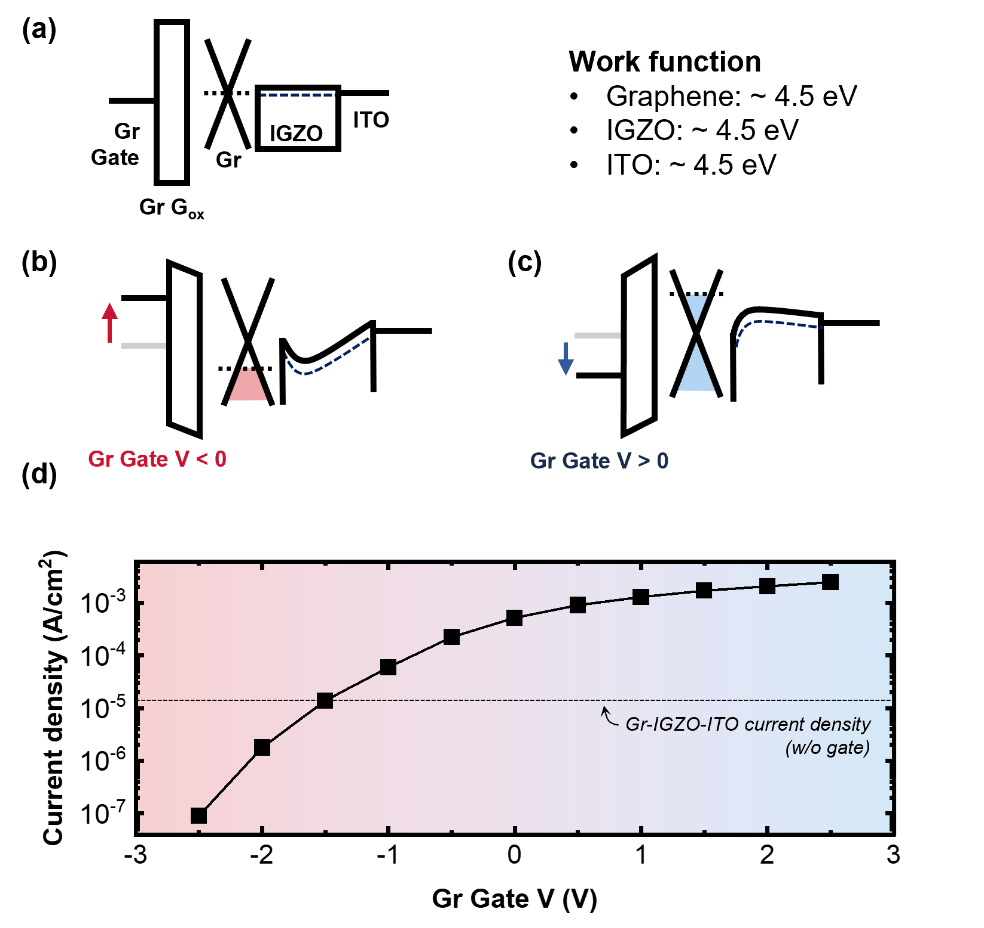


**Figure S3.** Electrical characteristics of graphene-IGZO interface. (a) Energy band diagram of gate, gate oxide, graphene, IGZO, and ITO at equilibrium. (b) Energy band under negative Gr-gate bias. (c) Energy band under positive Gr-gate bias. (d) Gr-gate controlled current density, compared with the reference current density without gate.

Figure S3 illustrates how the Fermi-level of graphene is modulated by the gate voltage and how this modulation influences the current density across the Gr–IGZO–ITO junction. As the gate voltage is swept from negative to positive values, a gradual increase in current density is observed, indicating a reduction in the contact barrier due to the upward shift of graphene’s Fermi level. Compared to the reference current measured without gate bias (dashed line), this trend confirms that electrostatic gating effectively modulates the graphene–IGZO interface, enhancing carrier injection and enabling precise control over device conduction.


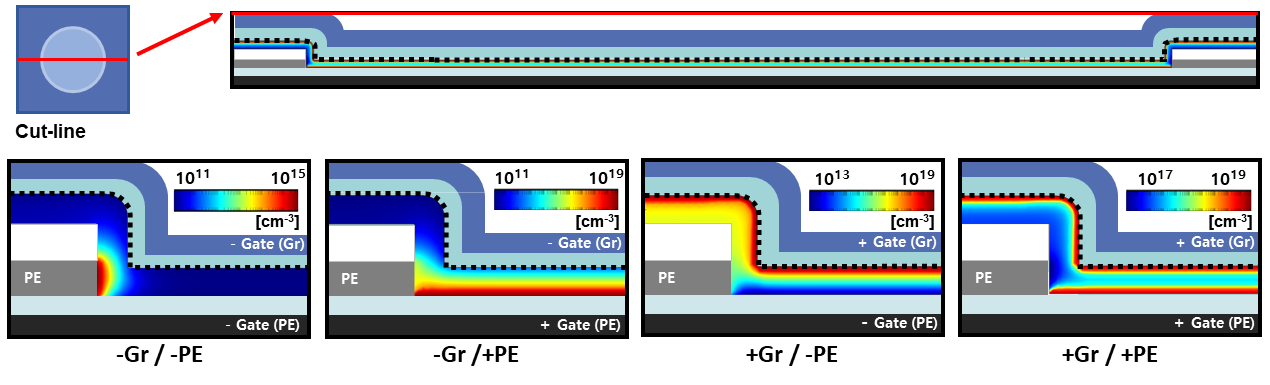


**Figure S4.** Simulation of carrier distribution in channel layer cross-sections for two gates combinations.

| **Channel** | **Channel length [nm]** | **Off current density** | **V_G_ range [V]** | **On/off ratio** | **Off current change vs V_DS_** | **Ref** |
| --- | --- | --- | --- | --- | --- | --- |
| MoS_2_ | 25 | 10^-2^ A/cm^2^  @V_DS_ 0.1V | 60  (Back gate SiO_2_ 300nm) | ~10^3^ | Increases | [32] |
| MoS_2_ | 4.5 | 10^-3^ A/cm^2^  @V_DS_ 0.1V | 60  (Back gate SiO_2_ 300nm) | ~10^6^ | Increases | [33] |
| IGZO | 4 | 10^-4^ A/cm^2^  @V_DS_ 0.1V | 60  (Back gate SiO_2_ 300nm) | ~10^5^ | Increases | [27] |
| MoS_2_ | 5 | 10^-1^ A/cm^2^  @V_DS_ 0.1V | 60  (Back gate SiO_2_ 300nm) | ~10^3^ | Increases | [20] |
| IGZO | 48 | 10^-4^ A/cm^2^  @V_DS_ 0.1V | 2  (ion gel) | ~10^4^ | Increases | [19] |
| WS_2_ | 10 | 10^-6^ A/cm^2^  @V_DS_ 0.1V | 8  (hBN 30nm) | ~10^6^ | Increases | [34] |
| IGZO | 40 | 10^-7^ A/cm^2^  @V_DS_ 0.1V to 2V | 2  (Dual HfO_2_ 20nm) | ~10^6^ | Negligible | This work |

**Table S1.** Comparison of electrical characteristics of graphene-based vertical transistors


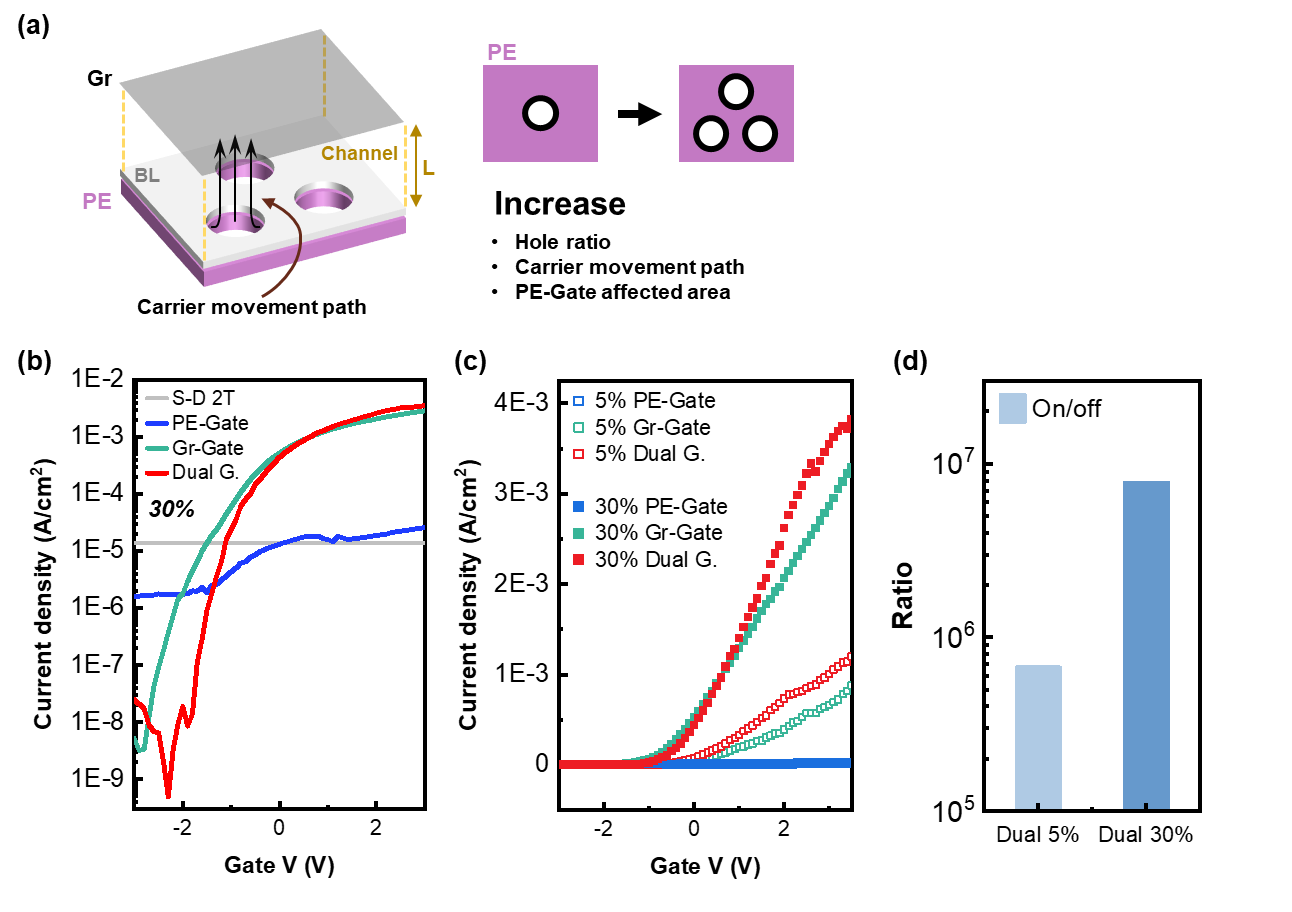


**Figure S5**. Comparison of electrical characteristics based on hole ratio and gate type (V_DS_ 0.1V). (a) Schematic illustrating carrier transport and effect of hole distribution. (b) Transfer curves of the transistor with 30% hole ratio. (c) Linear scale transfer characteristics of the transistor with 5% and 30% hole ratio. (d) On/off ratio of both devices.

Figure S5 illustrates the effects of gate modulation and micro-hole density on the electrical characteristics of vertical transistors. Figure S5a depicts the carrier transport pathways and hole distribution influencing factors. In this structure, micro-holes serve as key pathways for both electric field transmission and carrier injection. Figure S5b presents the transition characteristics of a device with a 30 % hole ratio under various gate modulation modes (floating, single-gate, and synchronized dual-gate operation). When both gates are driven simultaneously, the device exhibits a lower off-state current and a higher on-state current compared to the single-gate configuration, suggesting enhanced electrostatic control capability through dual modulation. Figures S5c and S5d compare devices with the same hole diameter (7 μm) but different hole ratios (5 % and 30 %). The higher hole ratio increases the total area available for field penetration and carrier injection, effectively expanding the channel region modulated by the gate. This results in improved field coupling in the off state and a wider injection path in the on state, leading to higher on-state current and an enhanced on/off current ratio. As shown in Figures S5 for different ratios, a higher hole ratio combined with dual modulation control achieves high output current and improved electrical characteristics.


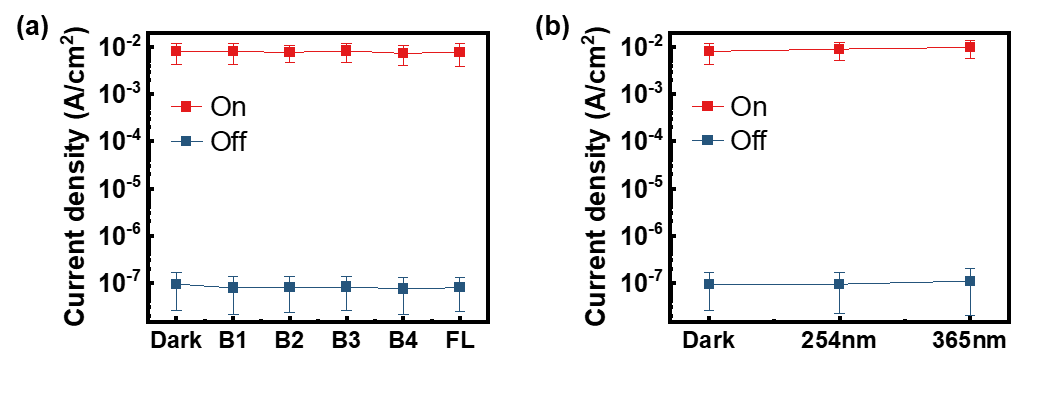


**Figure S6**. On-state and off-state drain currents measured under dual-gate operation for various illumination conditions at a drain voltage of V_DS_=0.1 V.
